# Postharvest Quality of Arugula (*Eruca sativa*) Microgreens Determined by Microbiological, Physico-Chemical, and Sensory Parameters

**DOI:** 10.3390/foods13193020

**Published:** 2024-09-24

**Authors:** Marina R. Komeroski, Thais Beninca, Keyla A. Portal, Patrícia S. Malheiros, Tâmmila V. Klug, Simone H. Flores, Alessandro O. Rios

**Affiliations:** 1 Postgraduate Program in Food Science, Institute of Food Science and Technology, Federal University of Rio Grande do Sul, Porto Alegre 90010-150, Brazil; thais_beninca@hotmail.com (T.B.); patricia.malheiros@ufrgs.br (P.S.M.); simone.flores@ufrgs.br (S.H.F.); alessandro.rios@ufrgs.br (A.O.R.); 2 Department of Nutrition, School of Medicine, Federal University of Rio Grande do Sul, Porto Alegre 90010-150, Brazil; keyla200215@gmail.com; 3 Postgraduate Program in Science and Food Technology, Department of Food Science, Farroupilha Federal Institute, Santa Maria 97050-685, Brazil; tammilaklug@gmail.com

**Keywords:** Brassicaceae microgreens, packaging, shelf-life assessment, microbiological analysis, physico-chemical parameters

## Abstract

(1) Background: Cultivating microgreens is emerging as an excellent market opportunity. Their easy, short, and sustainable production methods are the main reasons they are approved by growers. However, a feature that still prevents its further spread is the microbiological risk and their rapid senescence. The present study was conducted to evaluate the post-harvest storage and shelf life of arugula microgreens in different packaging through microbiological, physico-chemical, and sensory parameters; (2) Methods: Plants were stored at 5 °C in open air, vacuum sealed, and under modified atmosphere bags and tested at 0, 3, 5, 7, and 10 days; (3) Results: Microgreens stored in all packaging were safe for consumption within ten days. Regarding physical and chemical parameters, open packaging proved to be promising, with less weight loss and slower chlorophyll degradation. The sensory analysis demonstrated that the microgreens stored in the vacuum-sealed packaging showed a decrease in quality from the fifth day onwards for all attributes. However, the MAP presented good scores with a better visual quality, similar to the fresh microgreens.

## 1. Introduction

Arugula is one of the most consumed vegetables from the Brassicaceae family in the world [1,2] and the intensity of its aroma, pungency, and crunchiness seem to be decisive in consumer acceptance [3]. Microgreens of this vegetable are consumed fresh in salads but are also served as a garnish in other dishes, such as soups and sandwiches [4]. These plants contain significant amounts of important bioactive compounds and minerals [5], often being higher than adult plants of the same species [6], since they receive only light treatments [7] and are preceded by the germination stage [8]. This crop has a fast production cycle [9] and can be produced in greenhouses, in soil, or, more commonly, in soilless systems using solid organic or inorganic growing media or hydroponics [10], demonstrating the potential of these products to adapt the production of leafy vegetables to different scales [11]. If they are produced hydroponically, the soil is replaced by a substrate and seedlings are fed with a solution containing all the essential elements for their growth [12], allowing them to be grown organically [13]. Komeroski et al. [14] showed a high 45 protein, total fiber, and soluble fiber content of arugula microgreens grown using this system.

Choosing a culture medium with adequate microbiological characteristics is extremely important to ensure the safe consumption of microgreens, since the chosen medium may represent a contamination source [15]. For example, heat and humidity are the same ideal growth conditions for microgreens and pathogens such as *Salmonella, Listeria*, and *E. coli* O157:H7. These bacteria can infect seeds through small cracks and multiply to high levels during sprouting [16].

Before harvest, the greatest risks of contamination are related to irrigation water, substrate, and other factors [15]. In this regard, a delicate balance is required to maintain lower temperature (0–5 °C) and humidity conditions (50–85%) that optimizes the quality retention and microgreens shelf life while discouraging the growth of microbes and human pathogens [17]. However, data on potential microbiological hazards post-harvest are lacking. Currently, this type of product is packaged for subsequent sale without any disinfectant treatment, increasing consumer health risks.

According to Mir et al. [18], advances in packaging technology will help maintain the quality of microgreens for longer periods and extend their shelf life. In addition to quality parameters, functional information from these plants will help select the specific crop for a particular type of storage.

Modified atmosphere packaging (MAP) extends shelf life by ensuring persistent storage temperature and limited oxygen or moisture flows [19] by changing the gas composition, creating an appropriate atmosphere inside the packaging film, and effecting the integrity of the leaf tissue membrane [20]. Nowadays, it is one of the most effective technologies in maintaining the quality and extending the shelf life of fresh produce [21].

This work goal was to evaluate the post-harvest storage and shelf life of arugula microgreens in open, vacuum-sealed, and modified atmosphere packaging through microbiological, sensorial, and physico-chemical analysis.

## 2. Materials and Methods

### 2.1. Growing Conditions

Arugula (*Eruca sativa* L.) seeds were purchased from Isla Sementes^®^ Ltda. (Rio Grande do Sul, Brazil). The cultivation was carried out at the Laboratory of Bioactive Compounds of the Institute of Food Science and Technology of the Federal University of Rio Grande do Sul (ICTA/UFRGS) according to the methodology proposed by Di Gioia et al. [10], with some modifications:

Seeds were placed in Green-up^®^ phenolic foams with central holes and soaked in water for 48 h to promote germination according to the supplier’s recommendation. The cultivation system used was floating hydroponic. In this case, these sown foams were placed on top of perforated polystyrene boards and irrigated using a nutrient solution prepared with potable water containing (mg L^−1^) 6% nitrogen, 9% phosphorus, 29% potassium, 2.7% magnesium, 5% sulfur, 0.2% iron, 0.05% manganese, 0.02% zinc, 0.05% boron, 0.03% copper, 0.002% cobalt, 0.006% nickel, and 0.01% molybdenum. An air pump was used to oxygenate the nutrient solution. Seeded trays were kept at 25 °C in a controlled environment and were exposed to sunlight for a 12 h photoperiod.

Microgreens were harvested after 14 days of germination, cutting the plants manually with scissors a few millimeters above the phenolic foam.

The microgreens were packed (30 g) in polypropylene bags ((15 × 20 × 1.2 cm; OTR 50 cm^3^/m^2^/24 h/bar at 23 °C and 75% RH; residual pressure in the vacuum package was <100 Pa; 12 mm of thickness)-TecMaq^®^, São Paulo, Brazil) in different forms: open packaging (Open), the plastic bag remains with the upper side open; vacuum sealed packaging (Sealed), using the Fastvac^®^ sealer (model TM 250, TecMaq^®^, São Paulo, Brazil), and modified atmosphere packaging (MAP), in the same equipment, with the following gas composition: 5% carbon dioxide, 2.1% oxygen, and 92.9% nitrogen, as recommended by Chitarra and Chitarra [22]. They were then stored at 5 °C in a climate chamber (model NL-41-ALT, New Lab^®^, São Paulo, Brazil) under a 12-h photoperiod (light/dark cycle) for ten days. Sampling was performed after 3, 5, 7, and 10 days of storage. Three packs were sampled at each time and analyzed as independent replicates for shelf-life evaluation.

### 2.2. Microbiological Analysis

#### 2.2.1. *Salmonella* spp. and *Listeria monocytogenes* Analysis

The isolation of *Salmonella* spp. and *Listeria monocytogenes* in samples of microgreens stored in open, sealed, and modification atmosphere bags for 0, 5, and 10 days was carried out according to the ISO 6579-1:2017/Amd 1:2020 [23] and ISO 11290-1:2017 [24] methodology, respectively.

For *Salmonella* pre-enrichment, 10 g of the sample was added to 90 mL of buffered peptone water (BPW; HIMEDIA^®^) and incubated at 37 °C for 24 h. Subsequently, selective enrichment was carried out in Muller Kauffmann Tetrationate Broth (MKTTn; HIMEDIA^®^, Kelton, PA, USA) at 37 °C for 24 h and Rappaport–Vassilidis Soy Broth (RVS; HIMEDIA^®^) at 37 °C and 43 °C for 24 h. Then, seeding was carried out on Xylose Lysine Desoxycholate Agar (XLD; KASVI^®^, Paraná, Brazil) and Hektoen Enteric Agar (HE; HIMEDIA^®^) plates, both being incubated at 37 °C for 24 h [25]. Typical colonies were selected and subjected to identification through proteomic analysis using matrix-assisted laser desorption/ionization mass spectrometry and time of flight (MALDI-TOF/MS), model Autoflex Speed (Bruker Corporation, Bremen, Germany), following the method of Barbosa et al. [26].

For *Listeria* pre-enrichment, 10 g of the sample was added to 90 mL of Half-Fraser broth (HIMEDIA^®^) and incubated at 30 °C for 25 h. Subsequently, sowing was carried out on Listeria Ottaviani & Agosti (ALOA^®^) and OXFORD^®^ agar plates, incubating at 37 °C for 24 h to 48 h. Furthermore, 100 µL of the contents of the pre-enrichment tube were transferred to 10 mL of Fraser broth and incubated at 37 °C for 24 h. Afterward, seeding was carried out on ALOA^®^ and OXFORD^®^ plates incubated at 37 °C for 24 to 48 h [25]. All experiments were performed in duplicate.

#### 2.2.2. Total Enterobacteriaceae and *Escherichia coli* Count

Total Enterobacteriaceae and *Escherichia coli* in samples of microgreens stored in open air, sealed packaging, and under a modified atmosphere for 0 and 10 days was determined according to the Petrifilm^TM^ method [18]. Subsequently, the plates were incubated at 35 °C for 24 h to count total Enterobacteriaceae and 48 h to count *Escherichia coli* [25]. Experiments were performed in duplicate.

#### 2.2.3. Mesophilic, Psychrophilic, and Molds and Yeasts Count

The total count of mesophilic, psychrophilic, and molds and yeasts in samples of microgreens stored in open air, sealed packages, and under a modified atmosphere for 0, 5, and 10 days was determined according to the plating methods APHA 08:2015, APHA 13.61:2015, and APHA 21:2015, respectively. The following media and incubation conditions were used: Plate Count Agar (PCA) for mesophilic (35 °C for 48 h) and psychrotrophilic (7 °C for 10 days) and Dichloran Rose Bengal Chloramphenicol (DRBC) agar plates for molds and yeasts (25 °C for 5 days). The results were expressed as log CFU/g [25]. Experiments were performed in duplicate.

### 2.3. Physicochemical Analysis

#### 2.3.1. Color

Microgreen samples were measured using a colorimeter from Konica Minolta^®^-Osaka/Japan (model Chrona Meter CR400). This equipment identifies the color spectrum in a three-dimensional system (CIE standard illuminant D65 and standard observer 10), with the vertical axis, “L”, referring to the color of the sample from black to white; axis “a”, from green to red; and axis “b” from blue to yellow. The L-axis ranges from 0 to 100, with values over 50 being the lighter samples and below 50 the darker samples. All readings were performed in triplicate.

#### 2.3.2. pH, Soluble Solid Content and Titratable Acidity

Microgreens were ground in a tissue homogenizer Ultra Turrax IKA^®^ (model T25, IKA, São Paulo, Brazil) in a ratio of 1:5 (leaves:water) and then filtered, and its pH was measured at 25 °C using a Satra^®^ pH meter (model pHS-3E, Pró-Análise, Porto Alegre, Brazil). The Brix° were measured using an Atago^®^ refractometer (model PAL-3, Atago, São Paulo, Brazil) according to the AOAC methodology [27]. The titratable acidity (TA) was measured by titrating a 10 mL aliquot of microgreen juice extracts with 0.1 mol/L NaOH to the end point of pH 8.2. The results were expressed as the concentrations of (H^+^) in the unit of mol/L based on the fresh weight mass of the sample [28]. 

#### 2.3.3. Weight Loss

Weight loss was performed by scaling the microgreens before and after storage. Weight loss was computed according to the following formula [29]:

Weight loss (%) = (m_0_ − m_1_)/m_0_ × 100% (1)

where:

m_0_ = the weight (g) of microgreens before storage

m_1_ = the weight (g) of microgreens after storage

#### 2.3.4. Chlorophylls

The samples (0.4 g) were macerated with 10% (*w*/*v*) of 80% (*v*/*v*) acetone–water and then vortexed for 10 min. The homogenized tissue was centrifuged (5000× *g* for 15 min) and the supernatants were filtered. Samples were analyzed in UV–VIS spectrophotometer with the absorbance of the solution mixture at 647 nm for chlorophyll “a” and 663 nm for chlorophyll “b”, and the concentration was determined using the CHLs equations, according to method of Lichtenthaler and Wellburn [30]. The results were expressed on a dry basis.

### 2.4. Sensory Evaluation

Fresh (control, harvested on each day of analysis) and stored microgreens (3, 5, and 7 days) were submitted to sensory analysis at the Laboratory of Sensory Evaluation of the Food Science and Technology Institute (ICTA) of UFRGS to evaluate the following attributes: appearance, texture, taste, odor, color, and overall acceptance.

40 Adult judges of both genders (the average age was ±34 years, ranging from 18–49; 72.5% of the judges (n = 29) were female while 27.5% (n = 11) were male) were randomly and voluntarily recruited to receive a slice of approximately 1 g of each formulation, with three-digit random codes, a glass of water to clean the taste buds, and an evaluation sheet for sensory analysis, which contained a hedonic scale of nine points (1 = Dislike extremely, 9 = Like extremely). The analysis was carried out over a period of 20 min and the judges signed the Term of Informed Consent in accordance with the rules of research involving human beings.

This procedure was approved by the ethics and research committee of the UFRGS. Number of the Certificate of Presentation of Ethical Review: 65303222.5.0000.5347.

To calculate the acceptability index (AI) of each treatment (>70% = well accept), the following expression was used, as described by Viana [31]:

AI (%) = A × 100/B (1)(2)

where:

A = average grade obtained for each treatment

B = maximum grade given for each treatment

### 2.5. Statistical Analysis

Data regarding shelf life in different packaging were subjected to two-way analysis of variance ANOVA. The mean values were compared using Tukey’s test and separated by a least significant difference test (*p* < 0.05). Statistical analysis was performed using the software Statistica 14.0.1.

## 3. Results and Discussion

Microbiological analyses were performed to evaluate the contamination of the growing media and microbial growth levels on microgreens stored in different packaging. *Salmonella* spp. and *Listeria monocytogenes* were not observed in arugula microgreens, regardless of the day of storage and packaging used (Table 1). The same was observed by Priti et al. [32], who worked with mung bean (*Vigna radiata* L.), lentil (*Lens culinaris* subsp. culinaris), and Indian mustard (*Brassica juncea* L.) microgreens and did not detect *Salmonella* or *Listeria* in any of the samples tested.

Generally, mesophilic and psychrotrophic bacteria counts and enumeration of total Enterobacteriaceae are useful for indicating the shelf-life duration and microbial quality of foods [33]. Regarding the Enterobacteriaceae count (Table 2), the presence of these microorganisms was verified. Despite this, and in accordance with resolution (RDC 724/2022) and normative instruction (IN 161/2022) of the Brazil National Health Surveillance Agency [34,35] for microbiological food standards for fresh and prepared vegetable products, with no specific standard for microgreens, it proves good hygiene conditions and correct handling of samples during harvest and storage, regardless of the packaging used. We also observed the absence of *E. coli* on all samples.

Jablasone, Warriner, and Griffiths [36] found *E. coli* O157:H7 in the internal tissues of watercress, lettuce, radish, and spinach seedlings, but not in the mature plants’ tissues. The pathogen preferentially colonized the epidermal root junctions since, during seed germination, the seed releases a mixture of carbohydrates and peptides that can attract neighboring bacteria in the rhizosphere.

Chandra et al. [37] suggested that bacterial populations can easily grow on microgreens’ delicate and immature tissue structure and may be stimulated by sugars and other organic molecules derived from the endosperm breakdown during germination. Post-harvest, respiration becomes the primary physiological process of the plant, which uses its own previously accumulated metabolic reserves. However, depending on the intensity of biochemical reactions, tissues can reach senescence faster, becoming more susceptible to moisture loss and the development of microorganisms [22].

Table 3 shows the microbial population of arugula microgreens after 0, 5, and 10 days of storage. The total aerobic mesophilic bacteria during the initial phase of storage was 7.3 ± 0.22 log CFU/g, with a statistical difference (*p* < 0.05) for subsequent storage days. On day 10 of storage, we can observe a statistically significant difference (*p* < 0.05) in relation to the packaging used. This fact can be explained by fermentative activity, which is a characteristic of biological oxidations in an oxygen-free environment, such as vacuum-sealed packaging. In this environment, pyruvic acid is converted into carbon dioxide and acetaldehyde [15].

Similar values of total aerobic mesophilic bacteria were found in the work of Paradiso et al. [5] with chicory microgreens. During the initial storage phase, the number was 6.69 ± 0.07 log CFU per g and 8.19 ± 0.07 log CFU/g after 10 days of storage. In their work, psychrotrophic microorganism counts were very similar to those of mesophilic microorganisms, which matches this study at 5 and 10 days of storage.

Authors such as Chandra et al. [37] and Xiao et al. [38] identified the presence of mesophilic aerobic bacteria and molds and yeasts in these vegetables, with up to 10^7^ CFU/g^−1^ and 10^5^ CFU/g^−1^, respectively. These levels can be considered potentially dangerous, both for the food’s safety and its sensory quality and preservation capacity. It is worth mentioning that the International Commission on Microbiological Specifications for Foods (ICMSF) [33] states that foods with aerobic microorganism counts above 10⁶ CFU/g typically show noticeable signs of spoilage, such as off-smells, off-tastes, and changes in appearance.

The growth of yeasts and molds on buckwheat microgreens was relatively slow during the initial 8 days of storage (about 5.5 log CFU/g) and increased obviously from 8 to 12 days (up to 8.2 log CFU/g) storage in the study of Yan et al. [29]. Similar behavior was observed in the microgreens in this study, except for those in the open packaging, which did not differ statistically (*p* < 0.05).

According to Kyriacou et al. [39], the optimal conditions for microgreen growth are a pH between 6.56 and 7.54. Unfortunately, this is the same range for the development of neutrophil bacteria. Data in the study of Huang, Luo, and Nou [40] showed that the proliferation of *Salmonella* and *L*. *monocytogenes* was more significantly impacted by long-term suboptimal refrigeration or frequent temperature fluctuation than short-term terminal exposure to higher temperatures in fresh-cut cantaloupe.

The environmental conditions that influence the development of plants in a hydroponic system are light, temperature, and humidity [41]. The FDA [42] requires that all foods in the “Time and Temperature Control for Safety” be maintained at a temperature not exceeding 5 °C. Fresh-cut leafy green vegetables all belong to this category.

However, controlling climatic factors seems to be challenging for microgreens growers, who occasionally treated seeds with hydrogen peroxide before planting to mitigate the potential proliferation of mold and pathogens [43]. However, as reported by these authors, there is little evidence regarding the effectiveness of H_2_O_2_ for controlling microorganisms, including pathogens, on nonfood-contact and food-contact surfaces.

In Table 4 we show the physico-chemical evaluation of microgreens up to the tenth day of storage. In this work, we observed a drop in pH within seven days of storage, with a statistical difference (*p* < 0.05) between open and modified atmosphere packaging. This can be explained by the degradation of nitrogenous compounds present in the leaves of microgreens, releasing ammonia. Ammonia, when combined with water, resulting from cellular respiration, forms ammonium hydroxide, a weak base. However, the production of acids during cellular respiration often exceeds the production of bases, resulting in tissue acidification [22].

The increasing acidity at 5 days, which happened in this study, may be attributed to the biochemical conversion of fatty acids to acids over time [44]. After that, it is expected to decrease over time due to the plant’s physiological processes.

In this study, the effect of time on the increase in soluble solids (SS) occurred on days 5 and 7, whereas the interaction with the MAP did not differ significantly in 10 days of storage. This can be attributed to differential utilization of metabolites in by respiration getting influenced by the permeabilities of the packaging material to gasses in this package [44].

Leafy vegetables are highly susceptible to water loss after harvest, and respiration and other senescence-related metabolic processes are the primary cause of postharvest loss [45]. In this work, the weight loss increased with increasing storage time, with no statistical difference (*p* < 0.05) for 7 and 10 days, but with a difference for MAP. It was observed that although the weight loss was greater in the MAP, the microgreens presented a better visual quality compared to those in the open packaging, since contact with the environment caused dehydration in the microgreens closest to the opening.

In the work of Patil et al. [21], the post-harvest treatment of ascorbic acid + citric acid along with MAP of broccoli microgreens significantly (*p* < 0.05) suppressed the weight loss and helped to retain better firmness. According to Khan and Mittal [46], the efficiency of MAP depends upon multiple factors like appropriate gas composition, oxygen transmission rate (OTR), freshness, degree of processing of the product, product surface area, metabolism, respiration rate, microbial quality of produce, storage temperature, and relative humidity.

As Kou et al. [19] and Xiao et al. [38] explained, a favorable O_2_/CO_2_ balance and absence of anaerobic conditions that cause physiological damage to leaf tissue is required. When controlling these factors, the effect of storage temperature on the shelf life of microgreens appears to be more critical than the gas permeability of the packaging. At a storage temperature of 5 °C, the microgreens remained good for consumption for up to ten days of analysis.

Chlorophyll content is important for the health benefits it offers and has an effect on the appearance of the microgreens. Various shades of greenness in microgreens add to their aesthetic appeal [47]. In this work, the microgreens’ total chlorophyll content (Table 5) ranged from 31.03 ± 1.38 mg/100 g to 15.04 ± 2.43 mg/100 g at 10 days in MAP. In general, the decline in chlorophylls can be observed from the third day onwards, with a statistical difference (*p* < 0.05). Chandra et al. [37] observed that polypropylene films, due to the more significant accumulation of CO_2_, caused faster and more irreversible damage to the membrane compared to polyethylene, generating unpleasant odors. Furthermore, electrolyte leakage may contribute to faster yellowing, tissue senescence, and chlorophyll degradation of vegetables [38].

Similar values were found for total chlorophyll in the work of Kowitcharoen et al. [48], who analyzed some Brassicaceae microgreens such as Rat-tailed radish and red cabbage (36.61 and 39.79 mg/100 g, respectively). In the study of Ghoora, Hanldipur, and Srividya [49], the total chlorophyll of radish microgreens was 50.9 mg/100 g, but with a similar proportion of chlorophyll a and b (chlorophyll a approximately 2× higher) in relation to this work.

Color parameters are presented in Table 5. The L coordinate indicates lightness, which decreased significantly over the storage period after 3 days. The a* coordinate, denoting greenness, becomes less negative, signifying a decrease in ing samples. The b* value, representing yellowing, also showed a regressive incline over greenness over the storage period. However, no significant difference (*p* < 0.05) was observed in open packaging over the same storage period for the a* coordinate.

Katsenios et al. [50] reported that a bright green color corresponds to the high-quality index for microgreens of green vegetable species, and yellowing suggests the product’s quality deterioration. Green basil microgreens showed similar behavior in the work of Ciriello et al. [51]: luminosity (L; 48.06), greenness (a*; −17.30), and yellowness (b*; 28.92). El-Nakhel et al. [52] and Petropoulos et al. [53] reported that nutrient availability may affect the color of microgreens’ leaves and, thus, improve the visual quality of the final product.

According to sensory analysis results (Table 6), the sealed package showed a significant difference (*p* < 0.05) at 5 and 7 days in all attributes when compared to the third day. This result can be proven by the visual quality of the microgreens in this packaging, as shown in Supplementary Figure S1 (FS1). In contrast, we can observe a similarity between the open and MAP treatments (Supplementary Figure S2 (FS2)). In the study of Dhaka et al. [54], mustard microgreen was the first to undergo deterioration, and its visual quality showed some changes after four days.

In the descriptive analysis conducted in the work of Bafumo et al. [55], the evaluators identified rocket and watercress microgreens as the most astringent, standing out for pronounced bitterness and sourness, distinguishing them from the rest. Assessed microgreens evaluated by Caracciolo et al. [56] had consistently greater appearance scores than those concerning texture and flavor. Their statistical analysis indicated that the observed differences in microgreens acceptability depended on two main sensory dimensions experienced through the consumer test: astringency/sourness and bitterness.

**Figure 1:**
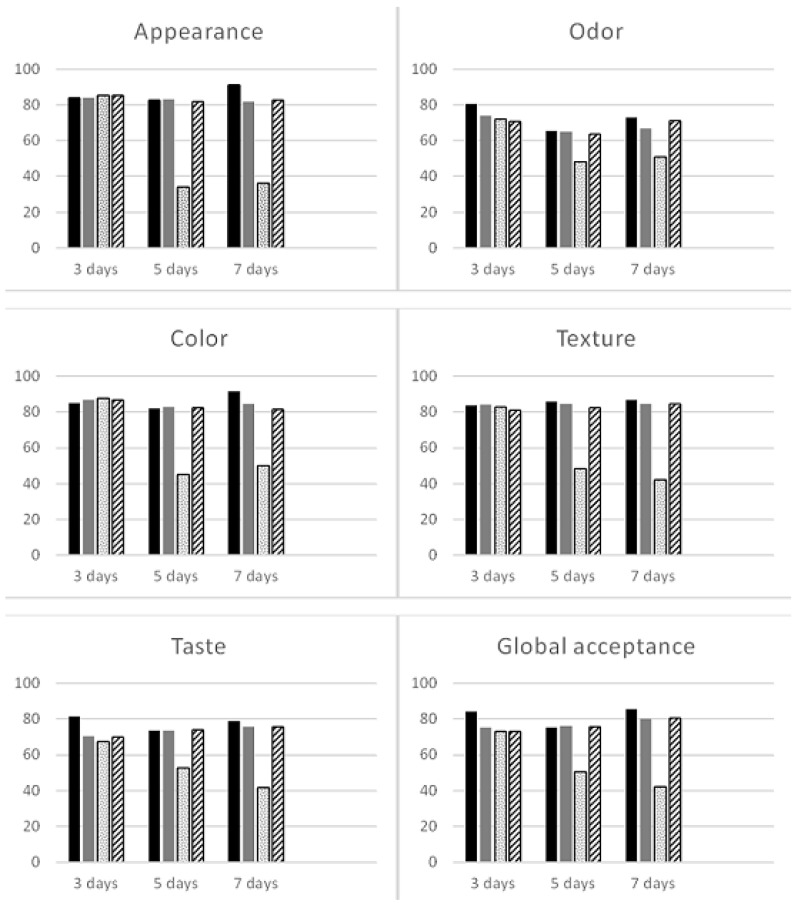
Acceptability index of sensory attributes of fresh (in black), open (in gray), sealed (with dots), and MAP (with stripes) arugula microgreens.

In relation to the acceptability index (Figure 1), as expected, the fresh sample obtained the best result (91.38%) for color, followed by appearance (91.11%) and texture (86.94%). It is noteworthy that regardless of the sealed packaging, at 5 and 7 days, all acceptability indexes of all treatments reached values above 60%, which is considered satisfactory.

## 4. Conclusions

Microgreens stored in all packaging types were safe for consumption over ten days. Regarding physico-chemical parameters, open packaging proved to be promising, with less weight loss and slower chlorophyll degradation, with maintenance of the greenness. The sensory analysis demonstrated that the microgreens stored in the vacuum-sealed packaging showed a worsening in quality from the fifth day onwards for all attributes. However, the MAP presented good scores, with a better visual quality, similar to fresh microgreens.

Further analysis can be conducted to enhance microgreens’ shelf life. We hope that the findings of the study will contribute to this field and inspire investigation into effective methods for food packaging and preservation.

## Figures and Tables

**Table 1:** Presence or absence of *Salmonella* spp. and *Listeria monocytogenes* in arugula microgreens stored in different packages for 0, 5, and 10 days.

Samples/Time	0 Days	5 Days	10 Days
*Salmonella* spp.			
Open	Absent	Absent	Absent
Sealed	Absent	Absent	Absent
MAP	Absent	Absent	Absent
*Listeria monocytogenes*			
Open	Absent	Absent	Absent
Sealed	Absent	Absent	Absent
MAP	Absent	Absent	Absent

Open: open packaging, the plastic bag remains with the upper side open; Sealed: vacuum sealed packaging; MAP: modified atmosphere packaging.

**Table 2:** Total Enterobacteriaceae and *Escherichia coli* (log CFU/g) in arugula microgreens stored in different packages for 0 and 10 days.

Samples (S)	Time (T)		*p* Value		
	0 Days	10 Days	S	T	S × T
	Total Enterobacteriaceae				
Open	7.61 ± 0.60 ^aA^	6.95 ± 0.44 ^bA^	0.862	0.039	0.862
Sealed	7.61 ± 0.60 ^aA^	7.22 ± 0.54 ^bA^			
MAP	7.61 ± 0.60 ^aA^	6.91 ± 0.38 ^bA^			
	*Escherichia coli*				
Open	Absent	Absent	-	-	-
Sealed	Absent	Absent	-	-	-
MAP	Absent	Absent	-	-	-

Open: open packaging, the plastic bag remains with the upper side open; Sealed: vacuum sealed packaging; MAP: modified atmosphere packaging. Different lowercase superscript in the same line indicates statistically significant difference for the time in the same package type by Tukey’s test (*p* < 0.05). Different uppercase superscript in the same column indicates statistically significant difference for the package in the same time by Tukey’s test (*p* < 0.05).

**Table 3:** Total count (log CFU/g) of mesophilic, psychrotrophilic, and molds and yeasts in arugula microgreens stored in different packages for 0, 5, and 10 days.

Samples (S)		Time (T)		*p* Value		
	0 Days	5 Days	10 Days	S	T	S × T
	Mesophilics					
Open	7.30 ± 0.22 ^aA^	7.40 ± 0.40 ^aA^	7.90 ± 0.26 ^aB^	0.008	<0.000	0.013
Sealed	7.30 ± 0.22 ^bA^	7.90 ± 0.23 ^bA^	8.80 ± 0.42 ^aA^			
MAP	7.30 ± 0.22 ^aA^	7.90 ± 0.37 ^aA^	7.70 ± 0.15 ^aB^			
	Psychrotrophs					
Open	5.70 ± 0.10 ^bA^	7.30 ± 0.16 ^aB^	7.90 ± 0.58 ^aA^	0.087	<0.000	0.003
Sealed	5.70 ± 0.10 ^cA^	7.60 ± 0.05 ^bAB^	8.60 ± 0.48 ^aA^			
MAP	5.70 ± 0.10 ^bA^	8.30 ± 0.50 ^aA^	7.70 ± 0.15 ^aA^			
	Yeasts and Molds					
Open	5.70 ± 0.06 ^aA^	6.50 ± 0.28 ^aB^	7.00 ± 1.09 ^aB^	0.002	<0.000	0.001
Sealed	5.70 ± 0.06 ^cA^	7.60 ± 0.09 ^bAB^	8.90 ± 0.53 ^aA^			
MAP	5.70 ± 0.06 ^cA^	8.30 ± 0.83 ^aA^	7.00 ± 0.01 ^bB^			

Open: open packaging, the plastic bag remains with the upper side open; Sealed: vacuum sealed packaging; MAP: modified atmosphere packaging. Different lowercase superscript in the same line indicates statistically significant difference for the time in the same package type by Tukey’s test (*p* < 0.05). Different uppercase superscript in the same column indicates statistically significant difference for the package in the same time by Tukey’s test (*p* < 0.05).

**Table 4:** Physicochemical analysis of arugula microgreens stored in different packages for 0, 3, 5, and 10 days.

Samples (S)	Time (T)					*p* Value		
	0 Days	3 Days	5 Days	7 Days	10 Days	S	T	S × T
pH								
Open	5.34 ± 0.10 ^bA^	6.39 ± 0.04 ^aA^	6.05 ± 0.53 ^aA^	4.54 ± 0.16 ^cA^	5.93 ± 0.10 ^aA^	0.432	<0.000	0.908
Sealed	5.34 ± 0.10 ^bA^	6.35 ± 0.11 ^aA^	6.24 ± 0.82 ^aA^	4.85 ± 0.11 ^cA^	6.29 ± 0.06 ^aA^			
MAP	5.34 ± 0.10 ^bA^	6.20 ± 0.07 ^aA^	6.12 ± 0.87 ^aA^	4.95 ± 0.12 ^cA^	6.26 ± 0.23 ^aA^			
Soluble solids								
Open	9.43 ± 0.05 ^bA^	9.26 ± 0.05 ^cB^	9.70 ± 0.00 ^aA^	9.66 ± 0.05 ^aA^	9.50 ± 0.00 ^bA^	0.114	<0.000	0.017
Sealed	9.43 ± 0.05 ^bA^	9.43 ± 0.05 ^bA^	9.70 ± 0.00 ^aA^	9.66 ± 0.05 ^aA^	9.46 ± 0.05 ^bA^			
MAP	9.43 ± 0.05 ^bA^	9.43 ± 0.05 ^bA^	9.66 ± 0.05 ^aA^	9.66 ± 0.05 ^aA^	9.56 ± 0.05 ^abA^			
Titratable acidity								
Open	2.46 ± 0.92 ^aA^	0.90 ± 0.17 ^bcA^	1.43 ± 0.66 ^bA^	0.63 ± 0.15 ^cA^	0.53 ± 0.32 ^cA^			
Sealed	2.46 ± 0.92 ^aA^	0.86 ± 0.30 ^bcA^	1.46 ± 0.89 ^bA^	0.66 ± 0.05 ^cA^	0.40 ± 0.10 ^cA^	0.988	<0.000	0.999
MAP	2.46 ± 0.92 ^aA^	1.03 ± 0.25 ^bcA^	1.50 ± 0.70 ^bA^	0.76 ± 0.15 ^cA^	0.23 ± 0.05 ^cA^			
Weight loss								
Open	-	8.93 ± 2.02 ^bA^	9.05 ± 2.09 ^bC^	15.03 ± 1.36 ^aB^	16.85 ± 3.63 ^aB^	<0.000	<0.000	<0.000
Sealed	-	7.15 ± 2.43 ^cA^	13.10 ± 1.00 ^bB^	15.02 ± 2.38 ^abB^	17.79 ± 1.35 ^aB^			
MAP	-	9.86 ± 0.34 ^dA^	16.44 ± 1.76 ^cA^	20.32 ± 1.90 ^bA^	24.14 ± 1.27 ^aA^			

Open: open packaging, the plastic bag remains with the upper side open; Sealed: vacuum sealed packaging; MAP: modified atmosphere packaging. Different lowercase superscript in the same line indicates statistically significant difference for the time in the same package type by Tukey’s test (*p* < 0.05). Different uppercase superscript in the same column indicates statistically significant difference for the package in the same time by Tukey’s test (*p* < 0.05).

**Table 5:** Chlorophyll and color analysis of arugula microgreens stored in different packages for 0, 3, 5, 7, and 10 days.

Samples (S)	Time (T)					*p* Value		
	0 Days	3 Days	5 Days	7 Days	10 Days	S	T	S × T
Chlorophyll a								
Open	19.67 ± 0.64 ^aA^	18.69 ± 0.84 ^aA^	17.47 ± 1.22 ^aA^	14.80 ± 1.26 ^bA^	12.38 ± 0.73 ^bA^	<0.000	<0.000	<0.000
Sealed	19.67 ± 0.64 ^aA^	13.96 ± 0.30 ^cB^	16.04 ± 1.22 ^bA^	14.08 ± 0.03 ^cA^	11.42 ± 0.68 ^dA^			
MAP	19.67 ± 0.64 ^aA^	17.62 ± 0.39 ^bA^	17.16 ± 0.24 ^bA^	10.88 ± 0.22 ^cB^	8.70 ± 0.18 ^dB^			
Chlorophyll b								
Open	11.35 ± 2.02 ^aA^	9.37 ± 1.60 ^bA^	8.59 ± 1.32 ^bA^	11.31 ± 2.24 ^bA^	10.00 ± 0.83 ^bA^	<0.000	<0.000	0.720
Sealed	11.35 ± 2.02 ^aA^	6.77 ± 1.07 ^bA^	6.33 ± 1.37 ^bA^	5.91 ± 0.07 ^bA^	6.88 ± 1.94 ^bA^			
MAP	11.35 ± 2.02 ^aA^	8.71 ± 0.42 ^bA^	6.17 ± 1.51 ^bA^	5.51 ± 0.37 ^bA^	6.34 ± 2.32 ^bA^			
Total chlorophyll								
Open	31.03 ± 1.38 ^aA^	28.07 ± 0.93 ^abA^	26.07 ± 0.15 ^bcA^	26.11 ± 3.50 ^bcA^	22.39 ± 0.52 ^cA^	<0.000	<0.000	<0.000
Sealed	31.03 ± 1.38 ^aA^	20.73 ± 1.21 ^bcB^	22.38 ± 0.91 ^bB^	19.99 ± 0.10 ^bcB^	18.31 ± 1.26 ^cB^			
MAP	31.03 ± 1.38 ^aA^	26.33 ± 0.32 ^bA^	25.79 ± 1.72 ^bA^	16.39 ± 0.37 ^cB^	15.04 ± 2.43 ^cB^			
Color L								
Open	53.41 ± 6.56 ^aA^	51.64 ± 1.17 ^bA^	44.30 ± 1.96 ^bcA^	44.62 ± 0.03 ^cA^	39.08 ± 0.02 ^cA^			
Sealed	53.41 ± 6.56 ^aA^	44.58 ± 5.67 ^bA^	41.50 ± 1.14 ^bcA^	41.90 ± 0.03 ^cA^	41.39 ± 0.03 ^cA^	0.222	<0.000	0.377
MAP	53.41 ± 6.56 ^aA^	45.35 ± 3.17 ^bA^	44.94 ± 0.09 ^bcA^	40.53 ± 0.01 ^cA^	39.83 ± 0.29 ^cA^			
Color a*								
Open	−14.82 ± 1.50 ^aA^	−11.23 ± 3.82 ^aA^	−12.39 ± 0.01 ^aA^	−11.12 ± 0.03 ^aB^	−9.93 ± 0.02 ^aC^	0.596	<0.000	0.029
Sealed	−14.82 ± 1.50 ^bA^	−11.82 ± 2.99 ^bA^	−13.86 ± 1.69 ^bA^	−11.08 ± 0.01 ^bB^	−6.43 ± 0.01 ^aA^			
MAP	−14.82 ± 1.50 ^cA^	−13.01 ± 2.10 ^cA^	−11.79 ± 0.05 ^bcA^	−7.75 ± 0.01 ^aA^	−9.15 ± 0.03 ^abB^			
Color b*								
Open	25.15 ± 1.20 ^aA^	22.35 ± 3.84 ^abA^	26.81 ± 0.45 ^aA^	18.66 ± 0.25 ^bcA^	15.52 ± 0.02 ^cA^	0.002	<0.000	<0.000
Sealed	25.15 ± 1.20 ^aA^	20.60 ± 2.84 ^abA^	22.49 ± 3.25 ^abA^	17.88 ± 0.03 ^bcB^	14.57 ± 0.02 ^cB^			
MAP	25.15 ± 1.20 ^aA^	24.18 ± 1.54 ^aA^	17.19 ± 0.05 ^bB^	15.56 ± 0.02 ^bcC^	14.71 ± 0.21 ^cB^			

Open: open packaging, the plastic bag remains with the upper side open; Sealed: vacuum sealed packaging; MAP: modified atmosphere packaging. Different lowercase superscript in the same line indicates statistically significant difference for the time in the same package type by Tukey’s test (*p* < 0.05). Different uppercase superscript in the same column indicates statistically significant difference for the package in the same time by Tukey’s test (*p* < 0.05).

**Table 6:** Sensory evaluation of arugula microgreens stored in different packages for 3, 5, and 7 days.

Samples (S)	Time (T)				*p* Value	
	3 Days	5 Days	7 Days	S	T	S × T
Appearance						
Fresh	7.55 ± 1.63 ^abA^	7.43 ± 1.50 ^bA^	8.20 ± 0.96 ^aA^	<0.00	<0.00	<0.00
Open	7.66 ± 1.54 ^aA^	7.46 ± 1.37 ^aA^	7.37 ± 1.39 ^aA^			
Sealed	7.63 ± 1.57 ^aA^	3.07 ± 1.72 ^bB^	3.27 ± 1.89 ^bB^			
MAP	7.60 ± 1.28 ^aA^	7.35 ± 1.42 ^aA^	7.42 ± 1.52 ^aA^			
Odor						
Fresh	6.63 ± 1.63 ^aAB^	5.89 ± 1.55 ^aA^	6.57 ± 1.66 ^aA^	<0.000	<0.000	0.009
Open	6.50 ± 1.90 ^aAB^	5.84 ± 1.49 ^aA^	6.00 ± 1.63 ^aA^			
Sealed	6.33 ± 1.86 ^aB^	4.33 ± 1.82 ^bB^	4.57 ± 2.12 ^bB^			
MAP	7.25 ± 1.31 ^aA^	5.74 ± 1.75 ^bA^	6.40 ± 1.56 ^bA^			
Color						
Fresh	7.80 ± 1.41 ^abA^	7.38 ± 1.42 ^bA^	8.22 ± 0.97 ^aA^			
Open	7.88 ± 1.48 ^aA^	7.43 ± 1.41 ^aA^	7.62 ± 1.35 ^aA^	<0.000	<0.000	<0.000
Sealed	7.80 ± 1.45 ^aA^	4.07 ± 1.97 ^bB^	4.50 ± 2.18 ^bB^			
MAP	7.62 ± 1.06 ^aA^	7.41 ± 1.39 ^aA^	7.32 ± 1.54 ^aA^			
Texture						
Fresh	7.50 ± 1.39 ^aA^	7.74 ± 1.31 ^aA^	7.82 ± 1.15 ^aA^			
Open	7.45 ± 1.56 ^aA^	7.61 ± 1.33 ^aA^	7.62 ± 1.35 ^aA^	<0.000	<0.000	<0.000
Sealed	7.26 ± 1.84 ^aA^	4.33 ± 2.46 ^bB^	3.80 ± 2.15 ^bB^			
MAP	7.58 ± 1.27 ^aA^	7.41 ± 1.44 ^aA^	7.60 ± 1.48 ^aA^			
Taste						
Fresh	6.32 ± 2.35 ^aB^	6.64 ± 2.06 ^aA^	7.07 ± 1.99 ^aA^			
Open	6.03 ± 2.29 ^aB^	6.64 ± 1.85 ^aA^	6.82 ± 2.06 ^aA^	<0.000	0.156	<0.000
Sealed	6.25 ± 2.48 ^aB^	4.74 ± 2.33 ^bB^	3.77 ± 2.42 ^bB^			
MAP	7.37 ± 1.46 ^aA^	6.66 ± 1.72 ^aA^	6.80 ± 1.84 ^aA^			
Global acceptance						
Fresh	6.76 ± 1.88 ^bAB^	6.79 ± 1.55 ^bA^	7.67 ± 1.26 ^aA^			
Open	6.55 ± 2.00 ^aB^	6.84 ± 1.64 ^aA^	7.20 ± 1.34 ^aA^	<0.000	0.002	<0.000
Sealed	6.56 ± 1.98 ^aB^	4.53 ± 2.24 ^bB^	3.80 ± 2.17 ^bB^			
MAP	7.60 ± 1.21 ^aA^	6.82 ± 1.47 ^bA^	7.25 ± 1.37 ^abA^			

Open: open packaging, the plastic bag remains with the upper side open; Sealed: vacuum sealed packaging; MAP: modified atmosphere packaging. Different lowercase superscript in the same line indicates statistically significant difference for the time in the same package type by Tukey’s test (*p* < 0.05). Different uppercase superscript in the same column indicates statistically significant difference for the package in the same time by Tukey’s test (*p* < 0.05).

## Data Availability

The original contributions presented in the study are included in the article/Supplementary Materials, further inquiries can be directed to the corresponding author.

## Supplementary Materials

The following supporting information can be downloaded at: https://www.mdpi.com/article/10.3390/foods13193020/s1, Figure S1: Visual quality of fresh (without packaging), open (open packaging, the plastic bag remains with the upper side open), sealed (vacuum sealed packaging) and MAP (modified atmosphere packaging) arugula microgreens in seven days of storage; Figure S2: Sensory analysis radar chart of fresh (without packaging), open (open packaging, the plastic bag remains with the upper side open), sealed (vacuum sealed packaging) and MAP (modified atmosphere packaging) arugula microgreens.
