# Supplementary material for: Postharvest Quality of Arugula (Eruca sativa) Microgreens Determined by Microbiological, Physico-Chemical, and Sensory Parameters
Source: Foods. 2024 Sep 24;13(19):3020. doi: 10.3390/foods13193020 (PMC11476110; doi:10.3390/foods13193020)
Supplement: Supplementary file 1 [file foods-13-03020-s001.zip › foods-3210181-supplementary.pdf]

Supplementary materials

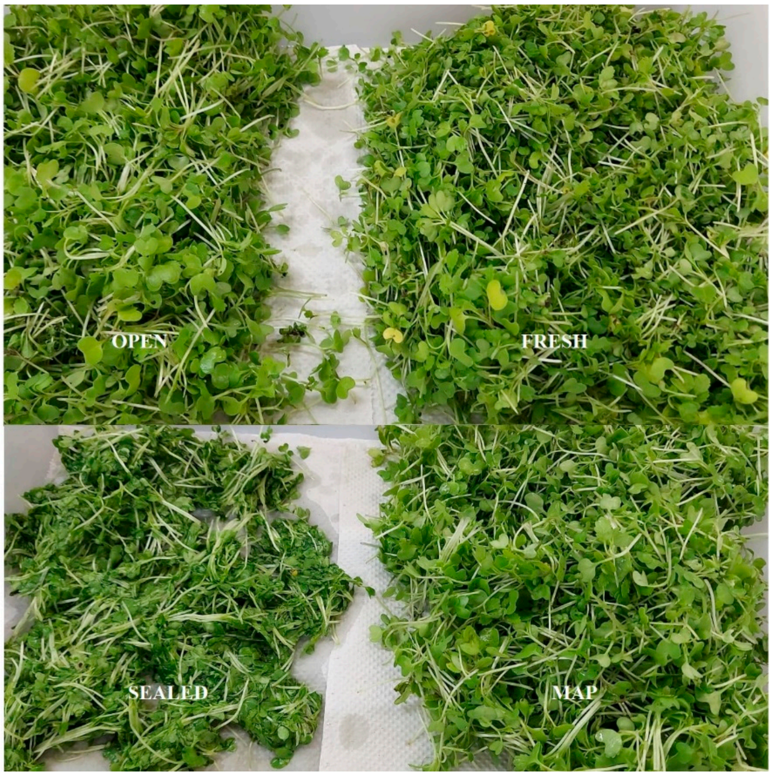

Figure S1. Visual quality of fresh (without packaging), open (open packaging, the plastic bag remains with the upper side open), sealed (vacuum sealed packaging) and MAP (modified atmosphere packaging) arugula microgreens in seven days of storage.

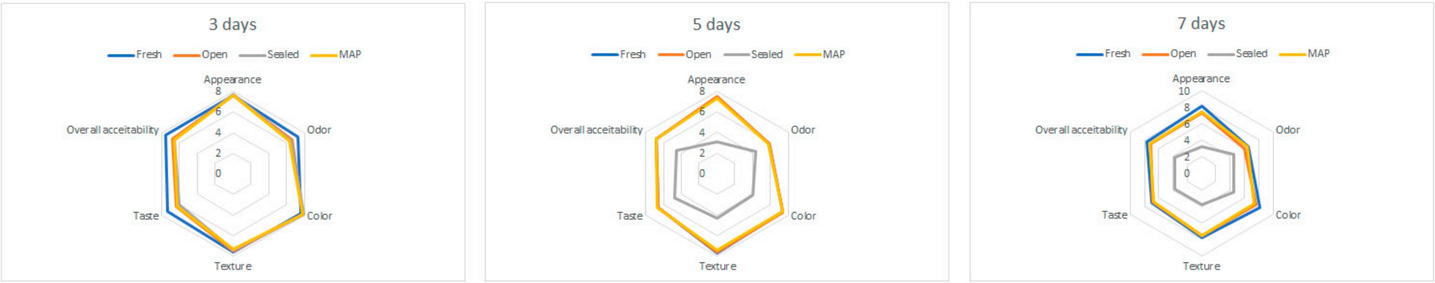

Figure S2. Sensory analysis radar chart of fresh (without packaging), open (open packaging, the plastic bag remains with the upper side open), sealed (vacuum sealed packaging) and MAP (modified atmosphere packaging) arugula microgreens.
